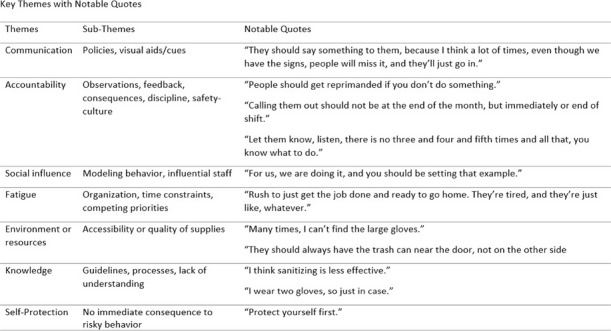# Impact evaluation of active surveillance testing for Candida auris on infection prevention behaviors

**DOI:** 10.1017/ash.2025.222

**Published:** 2025-09-24

**Authors:** Rachel Guran

**Affiliations:** 1 Memorial Healthcare System

## Abstract

**Background:** Candida auris (C. auris) is an emerging multidrug-resistant fungus associated with high mortality from healthcare-associated infections (HAIs). Public health authorities have classified it as an urgent threat due to its persistence in healthcare environments and difficulty in identifying asymptomatic carriers. Active surveillance testing (AST) is recommended to detect multidrug-resistant organisms (MDROs) early and evaluate transmission control measures. Infection prevention and control (IPC) strategies, including hand hygiene, personal protective equipment (PPE), and equipment disinfection, are essential for improving healthcare safety and ensuring continued prioritization by executive leadership. **Method:** A sequential explanatory mixed methods study evaluated the impact of AST for C. auris in a large South Florida hospital system. Retrospective data identified patient risk factors and calculated incidence and prevalence density rates. Logistic regression analyzed the association of risk factors with healthcare-onset C. auris events. Focus groups at a high-prevalence hospital ward explored barriers and facilitators to infection prevention behaviors, such as hand hygiene, PPE compliance, and equipment disinfection. The study applied the CDC Framework for Program Evaluation to assess AST’s influence on IPC behaviors to reduce C. auris transmission. **Results:** A total of 3,688 unique patients were included across five study hospitals, of which 185 (5%) patients were new positive C. auris cases. The study found an adjusted odds ratio of 3.279 (CI: 1.785-6.024) for patients admitted with tracheostomies for testing positive for C. auris on AST. The incidence density and prevalence of positive C. auris from AST and clinical results increased from 2020 to 2022 and decreased slightly in 2023. Percent positivity decreased from 7.58% in 2020 to 2.47% in 2023 as AST increased from 66 total to 1619 total per year. 40 staff participated in 6 individual focus groups separated by caregiver type and themes on IPC behaviors for C. auris emerged related to accountability, communication, social influence, self-protection, fatigue, environment or resources, and knowledge. Key barriers identified opportunities related to observations and feedback, perception of lack of discipline, and safety culture. Facilitators identified social influence and modeling as well as organization and accessibility of preferred supplies. **Conclusion:** While AST usage increased during the study period, the number of clinical and AST positive results decreased indicating early identification of cases had a possible impact on overall C. auris prevalence. AST as an intervention cannot be used effectively without a combined focus on IPC behaviors that are essential to preventing transmission of C. auris in the healthcare setting.

**Figure:**
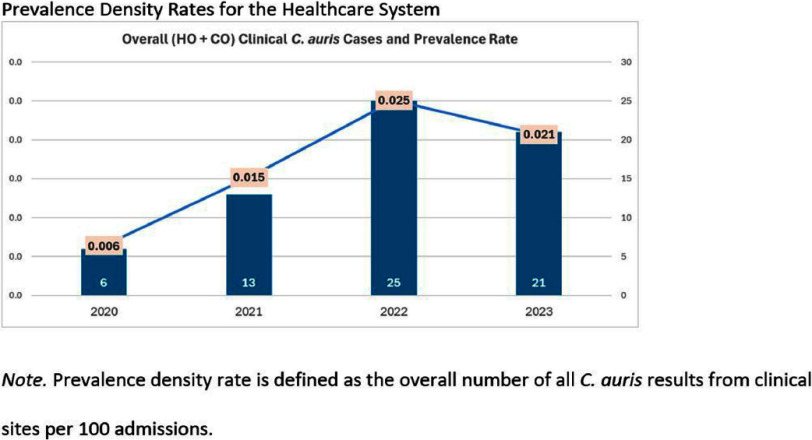

**Figure:**
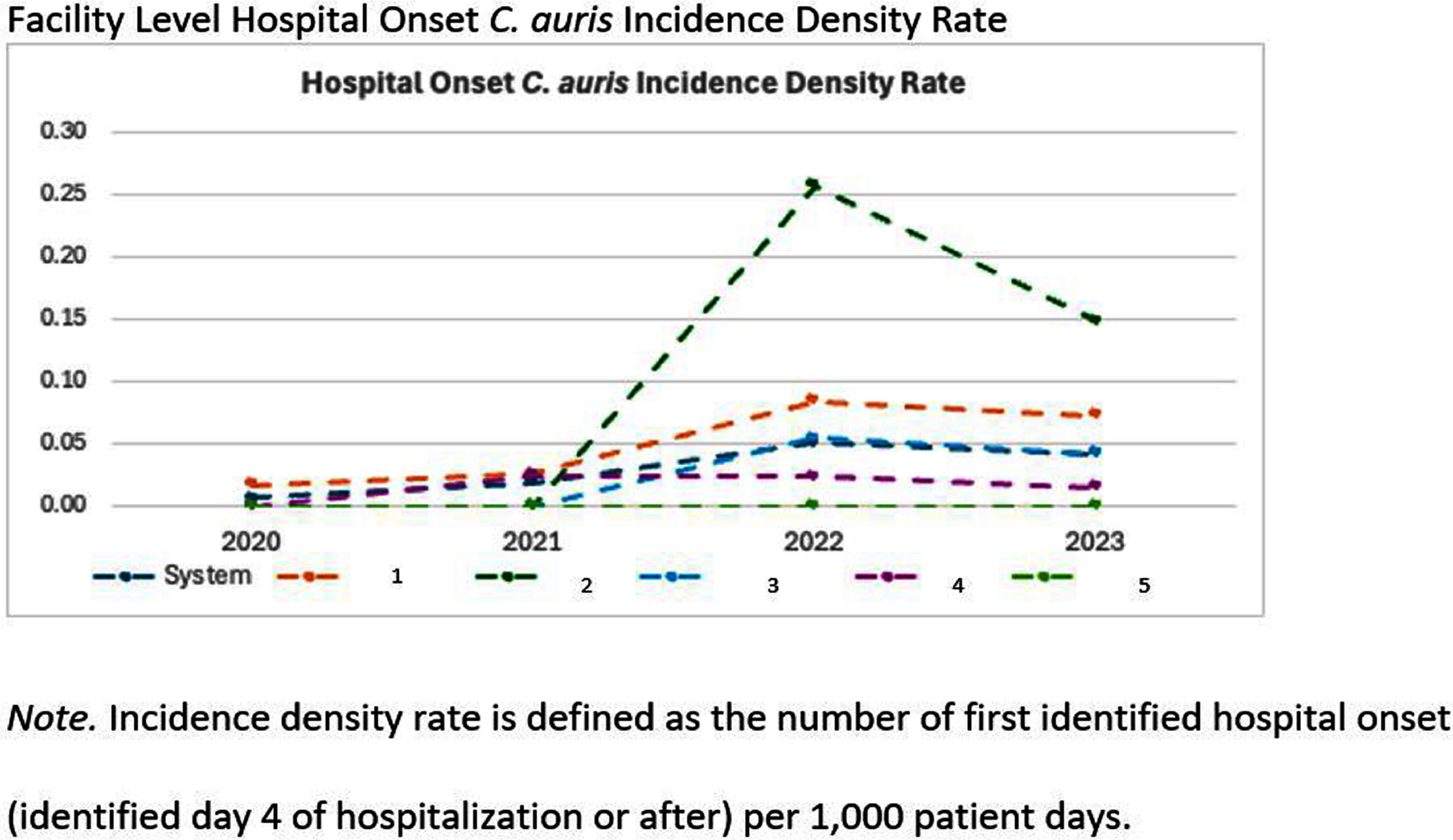

**Figure:**
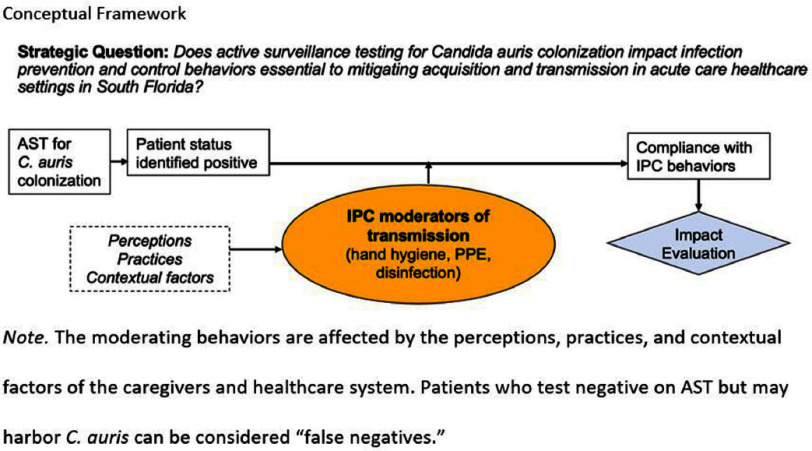

**Figure:**
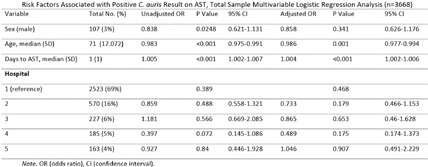

**Figure:**
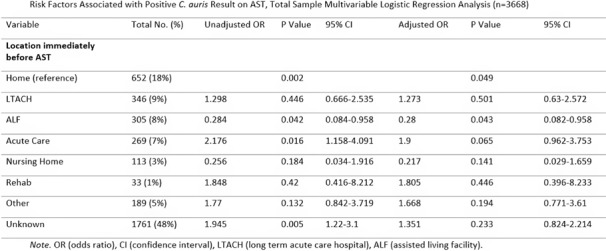

**Figure:**
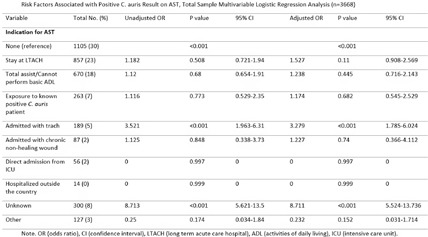

**Figure:**